# Chromosome-Scale Genome Assemblies of Aphids Reveal Extensively Rearranged Autosomes and Long-Term Conservation of the X Chromosome

**DOI:** 10.1093/molbev/msaa246

**Published:** 2020-09-23

**Authors:** Thomas C Mathers, Roland H M Wouters, Sam T Mugford, David Swarbreck, Cock van Oosterhout, Saskia A Hogenhout

**Affiliations:** 1 Department of Crop Genetics, John Innes Centre, Norwich Research Park, Norwich, United Kingdom; 2 Earlham Institute, Norwich Research Park, Norwich, United Kingdom; 3 School of Environmental Sciences, University of East Anglia, Norwich, United Kingdom

**Keywords:** Hemiptera, *Myzus persicae*, *Acyrthosiphon pisum*, insect genome assembly, synteny, sex chromosome, karyotype evolution

## Abstract

Chromosome rearrangements are arguably the most dramatic type of mutations, often leading to rapid evolution and speciation. However, chromosome dynamics have only been studied at the sequence level in a small number of model systems. In insects, Diptera and Lepidoptera have conserved genome structure at the scale of whole chromosomes or chromosome arms. Whether this reflects the diversity of insect genome evolution is questionable given that many species exhibit rapid karyotype evolution. Here, we investigate chromosome evolution in aphids—an important group of hemipteran plant pests—using newly generated chromosome-scale genome assemblies of the green peach aphid (*Myzus persicae*) and the pea aphid (*Acyrthosiphon pisum*), and a previously published assembly of the corn-leaf aphid (*Rhopalosiphum maidis*). We find that aphid autosomes have undergone dramatic reorganization over the last 30 My, to the extent that chromosome homology cannot be determined between aphids from the tribes Macrosiphini (*Myzus persicae* and *Acyrthosiphon pisum*) and Aphidini (*Rhopalosiphum maidis*). In contrast, gene content of the aphid sex (X) chromosome remained unchanged despite rapid sequence evolution, low gene expression, and high transposable element load. To test whether rapid evolution of genome structure is a hallmark of Hemiptera, we compared our aphid assemblies with chromosome-scale assemblies of two blood-feeding Hemiptera (*Rhodnius prolixus* and *Triatoma rubrofasciata*). Despite being more diverged, the blood-feeding hemipterans have conserved synteny. The exceptional rate of structural evolution of aphid autosomes renders them an important emerging model system for studying the role of large-scale genome rearrangements in evolution.

## Introduction

Mutation generates genomic novelty upon which natural selection and genetic drift can act to drive evolutionary change (Charlesworth 2009; Lynch et al. 2016; Charlesworth and Charlesworth 2017; Good et al. 2017). Primarily, sequence-level studies of genome evolution have focused on single-nucleotide polymorphisms and small indels. However, with the advent of long-read sequencing and other technologies that capture long-range linkage information, we are now able to study the effects of larger mutational events such as segmental duplications, deletions, and other complex structural variants (e.g., Chakraborty et al. 2018; Kronenberg et al. 2018). Chromosomes may undergo extensive rearrangement via inversions, translocations, fusions, and fissions (Eichler and Sankoff 2003). These macromutations can have dramatic consequences by altering gene regulation (Farré et al. 2019; Stewart and Rogers 2019) and modifying local recombination rates (Farré et al. 2013; Martin et al. 2019), and they are implicated in key evolutionary processes such as adaptation and speciation (Rieseberg 2001; Kirkpatrick and Barton 2006; Chang et al. 2013; Guerrero and Kirkpatrick 2014; Fuller et al. 2019; Wellband et al. 2019). Chromosome-scale genome sequencing and assembly are required to study such macromutations, and recent advances in genome assembly have reinvigorated the field (e.g., Dudchenko et al. 2017; Bracewell et al. 2019, 2020; Schield et al. 2019; Tandonnet et al. 2019; Teterina et al. 2020). So far, in insects, these studies have been restricted to a few holometabolous groups, such as Diptera (mainly *Drosophila* and mosquitoes) and Lepidoptera (butterflies) that have been the focus of concerted genome sequencing efforts.

Comparative genomics of Diptera and Lepidoptera has revealed conservation of whole chromosomes or chromosome arms (i.e., macrosynteny) over substantial periods of time. For example, tephritid fruit flies have maintained chromosome arms, known as Muller elements (Schaeffer 2018), over at least 60 million years (My) (Sved et al. 2016). Conservation of chromosome structure is even more striking in mosquitos, where chromosome arms have been maintained for at least 150 My despite substantial changes in genome size (Dudchenko et al. 2017). Among Lepidoptera, the ancestral chromosome complement has largely been maintained over 140 My, and where changes in karyotype have occurred, they have been driven by chromosome fusion and fission events that maintain ancestral chromosome fragments (d’Alençon et al. 2010; Heliconius Genome Consortium et al. 2012; Ahola et al. 2014; Davey et al. 2015). The green-veined white butterfly (*Pieris napi*) appears to be one of the few lepidopteran exceptions, as a chromosome-scale reference genome for this insect has recently revealed extensive genome rearrangement despite having a chromosome number similar to model species (Hill et al. 2019).

Nonetheless, chromosome number is highly variable across insects as a whole (Blackmon et al. 2017), suggesting that the conserved genome structures of Diptera and Lepidoptera cannot be used as models for all insects. A dramatic example of this can be found in aphids—an important group of hemimetabolous sap-sucking plant pests belonging to the insect order Hemiptera—where characterized karyotypes vary from 2*n* = 4 (two pairs of diploid chromosomes) to 2*n* = 72 (Blackman 1980). This variation occurs between closely related species, and even within species, suggesting a high rate of chromosome evolution (Blackman 1971; Panigrahi and Patnaik 1991; Blackman et al. 2000; Monti et al. 2012; Mandrioli et al. 2014; Manicardi, Nardelli, et al. 2015).

Aphid chromosome structure and life cycle may contribute to the rapid evolution of diverse karyotypes (Blackman 1980). First, aphids and other Hemiptera have holocentric chromosomes that lack localized centromeres (Hughes-Schrader and Schrader 1961; Melters et al. 2012; Drinnenberg et al. 2014). Instead, spindle fibers attach diffusely across the chromosome during meiosis and mitosis (Ris 1942, 1943). As such, both products of a chromosomal fission event can undergo replication, whereas in species with localized centromeres, the fragment lacking the centromere would be lost (Ris 1942; Schrader 1947). Second, aphids have an unusual reproductive mode—cyclical parthenogenesis—where they reproduce clonally via apomictic parthenogenesis during the spring, summer, and autumn, followed by a sexual stage that produces overwintering eggs from which asexually reproducing females hatch (Dixon 1977). Clonal lineages can persist for long periods without sexual reproduction and some species have become obligately asexual (Moran 1992; Simon et al. 2002). These bouts of prolonged asexuality, combined with males being derived from an asexual lineage, may enable rearranged karyotypes to persist and potentially contribute to speciation events, thus facilitating the evolution of diverse karyotypes.

Genome sequencing of a small number of aphid species has also revealed dynamic patterns of genome evolution, with extensive gene duplication having occurred throughout aphid diversification (IAGC 2010; Mathers et al. 2017; Thorpe et al. 2018; Li et al. 2019; Fernández et al. 2020; Julca et al. 2020). However, at the time this study started, aphid genome assemblies were highly fragmented (although see Li et al. [2019] and Chen et al. [2019]) and chromosome-scale genome assemblies had not yet been analyzed to assess the evolution of aphid karyotypes and how this compares with diverse Hemiptera.

Here, we generated high-quality chromosome-scale genome assemblies of two extensively studied aphid species: the green peach aphid *Myzus persicae*, a model generalist aphid and major crop pest (Mathers et al. 2017), and the pea aphid *Acyrthosiphon pisum*, a model for speciation genomics and basic aphid biology (Hawthorne and Via 2001; Brisson and Stern 2006; Peccoud et al. 2009; Pecoud and Simon 2010; Nouhaud et al. 2018). Comparison of these new aphid assemblies with a previously published chromosome-scale assembly of the corn-leaf aphid *Rhopalosiphum maidis* (Chen et al. 2019) showed that, over the last ∼30 My, aphid autosomes have undergone dramatic reorganization. In contrast, gene content of the aphid sex (X) chromosome remained unchanged.

While this work was under review (Mathers et al. 2020b), Li et al. (2020) also found extensive autosome reorganization in aphids by comparing *A. pisum* and *R. maidis* genomes and provided evidence that chromosome evolution of aphids is distinct from that of a psyllid, an obligate sexually reproducing species that, like aphids, belongs to the suborder Sternorrhyncha, within Hemiptera. In this study, we extend the analyses of hemipteran genome evolution beyond Sternorrhyncha by including the recently released chromosome-scale assemblies of *Rhodnius prolixus* (obtained from the DNA Zoo; Dudchenko et al. 2017) and *Triatoma rubrofasciata* (Liu et al. 2019), two blood-feeding heteropterans with obligate sexual life cycles whose divergence from Sternorrhyncha represents a basal split in extant Hemiptera (Johnson et al. 2018). By comparing across Hemiptera, we find evidence to support the ancient conservation of hemipteran X chromosome gene content and reveal divergent patterns of autosome evolution between aphids and the two investigated Heteroptera. Furthermore, using our new high-quality genome assemblies of *M. persicae* and *A. pisum*, we investigate the evolution and genome-wide distribution of aphid transposable elements (TEs), finding an association between the accumulation of specific repeat classes and autosomal synteny breakpoint regions as well as revealing new insights into aphid X chromosome dynamics.

## Results and Discussion

### Chromosome-Scale Assemblies of the *M. persicae* and *A. pisum* Genomes

**Fig. 1.**
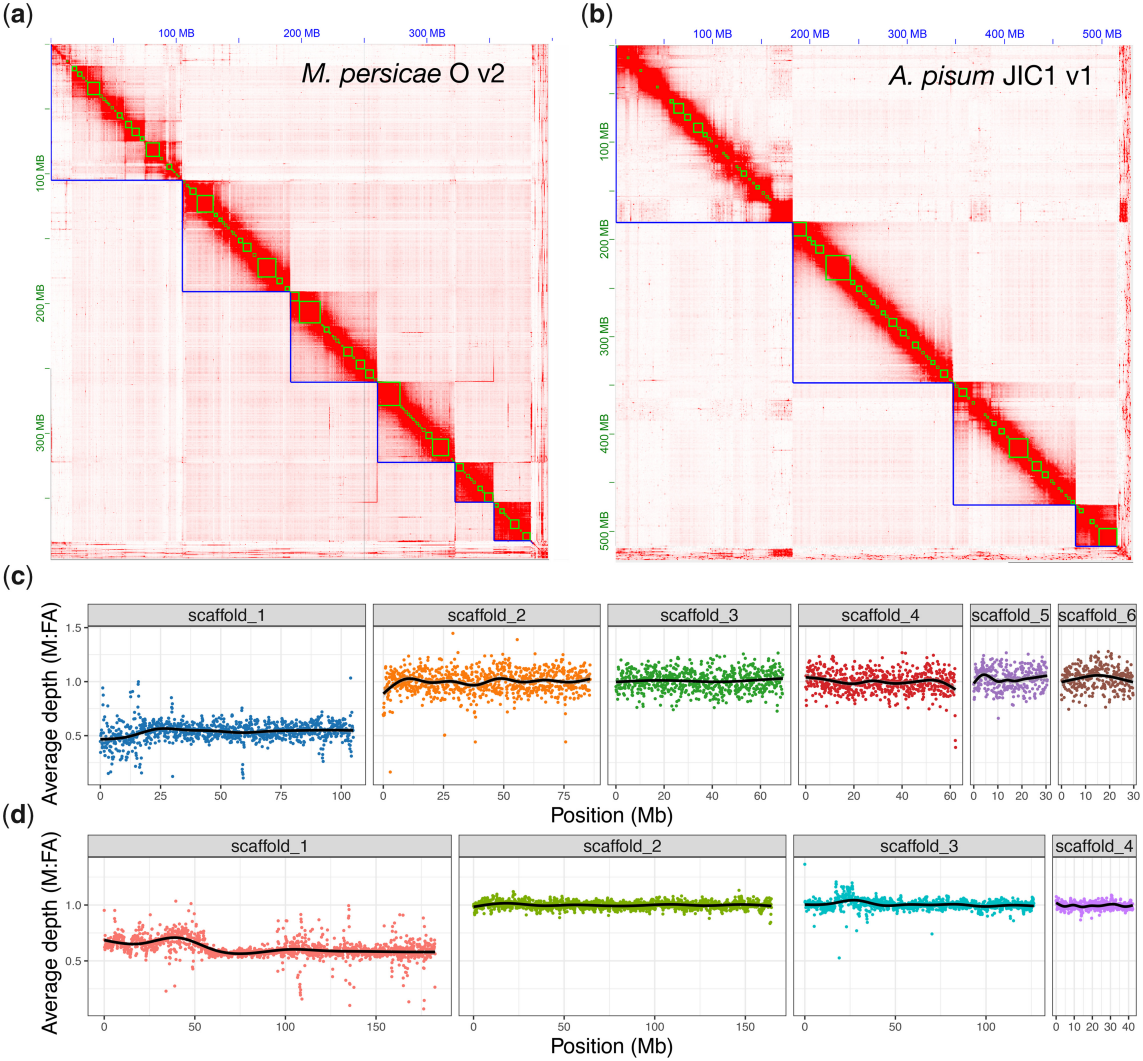
Chromosome-scale genome assemblies of *Myzus persicae* and *Acyrthosiphon pisum*. (*a*) Heatmap showing frequency of HiC contacts along the *M. persicae* clone O v2 (MperO_v2) genome assembly. Blue lines indicate super scaffolds and green lines show contigs. Genome scaffolds are ordered from longest to shortest with the *x* and *y* axis showing cumulative length in millions of base pairs (Mb). (*b*) As for (*a*) but showing HiC contacts along the *A. pisum* JIC1 v1 (ApisJIC1) genome assembly. In this instance, green lines indicate corrected scaffolds from the input assembly which was scaffolded with 10X Genomics linked reads prior to chromosome-scale scaffolding with HiC. (*c*) Male (M) to asexual female (FA) coverage ratio of *M. persicae* clone bisulfite sequencing genomic reads in 100-kb fixed windows across MperO_v2 chromosome-length scaffolds. The black line indicates the LOESS smoothed average. (*d*) As for (*c*) but showing the M to FA coverage ratio of *A. pisum* clone AL4 genomic reads across ApisJIC1 chromosome-length scaffolds.

High-quality, chromosome-scale, genome assemblies of *M. persicae* (clone O) and *A. pisum* (clone JIC1) were generated using a combination of Illumina short-read sequencing, Oxford Nanopore long-read sequencing, 10X Genomics linked reads (for *A. pisum*), and in vivoin vivo chromatin conformation capture (HiC) (fig. 1*a* and *b*). These new genome assemblies provide significant increases in contiguity compared with previously published assemblies at both the contig and scaffold level (table 1 and supplementary fig. 1, Supplementary Material online). For *M. persicae*, we report the first chromosome-scale genome assembly of this species with 97% of the assembled content contained in six scaffolds corresponding to the haploid chromosome number of this species (Blackman 1980). Compared with the original assembly of *M. persicae* clone O (Mathers et al. 2017), contig number is reduced from 23,616 to 915 and contig N50 is increased by 707% (59 kb vs. 4.17 Mb). For *A. pisum*, 98% of the assembled content was placed into four scaffolds corresponding to the haploid chromosome number of this species (Blackman 1980). Compared with a recently rescaffolded reference assembly of *A. pisum* dubbed AL4 (Li et al. 2019), we place an additional 14% (98% vs. 86%) of the *A. pisum* genome into chromosomes, reduce the number of contigs from 68,186 to 2,298, and increase contig N50 by 1,667% (0.03 Mb vs. 0.53 Mb). K-mer analysis of each assembly versus Illumina short reads shows very low levels of missing content and the absence of erroneously duplicated content due to the inclusion of haplotigs (allelic variation assembled into separate scaffolds) (supplementary fig. 2*a* and *b*, Supplementary Material online). Additionally, our *M. persicae* and *A. pisum* genome assemblies are accurate at the gene level, containing 94% and 98% of conserved Arthropoda benchmarking universal single-copy orthologs (BUSCO) genes (*n* = 1,066) as complete, single copies, respectively (supplementary fig. 3, Supplementary Material online). Therefore, the new assemblies of *M. persicae* and *A. pisum* are contiguous, accurate, and complete.

**Table 1.** Genome Assembly and Annotation Statistics for *Acyrthosiphon pisum*, *Myzus persicae*, and *Rhopalosiphum maidis*.

Species	*A. pisum*	*A. pisum*	*A. pisum*	*M. persicae*	*M. persicae*	*R. maidis*
Assembly	LSR1 v2	AL4 v1	JIC1 v1	O v1.1	O v2	BTI-1 v1
Sequencing approach^a^	S + IL + MP	HiC^b^	10X + ONT + HiC	IL + MP	IL + ONT + HiC	IL + PB + HiC
Base pairs (Mb)	541.68	541.12	525.80	354.7	395.14	326.02
% Ns	7.71	7.65	0.08	3.26	0.10	0.01
Number of contigs^c^	60,596	68,186	2,298	23,616	915	960
Contig N50 (Mb)^c^	0.03	0.03	0.53	0.06	4.17	9.05
Number of scaffolds	23,924	21,919	558	13,407	360	220
Scaffold N50 (Mb)	0.52	132.54	126.6	0.16	69.48	93.3
% of assembly in chromosome-length scaffolds	0	85.96	98.20	0	97.06	98.37
Protein-coding genes	36,939		30,784	18,433	27,663	17,629
Transcripts	36,939		34,135	30,247	31,842	17,629
Reference	IAGC (2010)	Li et al. (2019)	This study	Mathers et al. (2017)	This study	Chen et al. (2019)

a S, Sanger; IL, Illumina short reads; MP, Illumina mate-pairs; 10X, 10X Genomics linked reads; HiC, high-throughput chromatin conformation capture; ONT, Oxford Nanopore long reads; PB, PacBio long reads.

b In vitroIn vitro (Dovetail Chicago) and in vivo HiC used to correct and scaffold LSR1 v2.

c Scaffolds split on runs of 10 or more Ns.

Using our improved *M. persicae* and *A. pisum* genome assemblies, we annotated protein-coding genes in each species using evidence from RNA-seq data. For *M. persicae*, we aligned 160 Gb of RNA-seq data derived from whole bodies of unwinged (apterous) asexual females, winged asexual females, winged males and nymphs, and annotated 27,663 protein-coding genes. For *A. pisum*, we annotated 30,784 protein-coding genes, incorporating evidence from 23 Gb of RNA-seq data that were also derived from multiple morphs including unwinged asexual females, sexual females and males. The completeness of the annotations reflected that of the genome assemblies, with 93% and 92% of conserved Arthropoda BUSCO genes (*n* = 1,066) found as complete, single copies, in the *M. persicae* and *A. pisum* annotations, respectively (supplementary fig. 4, Supplementary Material online).

Protein-coding gene counts for our new annotations of *A. pisum* and *M. persicae* differ from previous versions with 6,155 fewer genes annotated in *A. pisum* JIC1 compared with LSR1 v2 and 9,230 more genes annotated in *M. persicae* clone O v2 compared with v1.1 (table 1). This is not entirely unexpected as gene counts can vary substantially depending on the gene annotation strategy used (Yandell and Ence 2012; Denton et al. 2014). Indeed, our gene counts are much closer to the independent annotations of *A. pisum* LSR1 v2 and *M. persicae* clone G006 v2 carried out by Thorpe et al. (2018), who used the same annotation pipeline employed in this study (BRAKER [Hoff et al. 2016, 2019]) and found 27,676 and 25,726 genes in *A. pisum* and *M. persicae*, respectively. Additionally, in the case of *M. persicae*, the use of additional RNA-seq data from diverse morphs sequenced for this study and elsewhere (Mathers et al. 2019) may have contributed to the discovery of additional genes. Finally, our improved genome assemblies may also contribute to the observed differences in gene count. The JIC1 v1 assembly of *A. pisum* is 15 Mb smaller than the LSR1 and AL4 assemblies (table 1) and is closer to the predicted *A. pisum* genome size (514 Mb; Wenger et al. 2020). In contrast, *M. persicae* clone O v2 contains an additional 40 Mb of sequence compared with v1.1 (table 1) and is also much closer to the predicted *M. persicae* genome size (409 Mb; Wenger et al. 2020).

### Identification of the Aphid Sex (X) Chromosome

To identify the X chromosome, we aligned the genomic DNA Illumina reads derived from asexual female and male morphs and calculated the male to asexual female coverage ratio in 100-kb fixed windows along each chromosome. Because sex is determined by random loss of one copy of the X chromosome in aphids (Wilson et al. 1997), with males carrying a single copy of the X chromosome, males should have half the coverage of females for the X chromosome and equivalent coverage for autosomes (Jaquiéry et al. 2018). In agreement with cytological analysis of *M. persicae* and *A. pisum* (Manicardi, Mandrioli, et al. 2015), we find that the longest scaffold in their respective assemblies has the expected coverage pattern of an X chromosome along its full length (fig. 1*c* and *d*). The remaining chromosomes do not deviate from the expected male to asexual female coverage ratio of 1:1, indicating an absence of X chromosome–autosome chimeras. Alignment of *A. pisum* JIC1 with the AL4 assembly and a previously published microsatellite linkage map (Jaquiéry et al. 2014) also confirms the identity of the *A. pisum* X chromosome as scaffold 1 and, overall, JIC1 v1 is in broad agreement with AL4 with the exception of a possible inversion at the beginning of scaffold 3 that may represent true biological variation or an assembly error in JIC1 v1 (supplementary fig. 5, Supplementary Material online). Importantly, we assemble and place an additional 50 Mb of the X chromosome in the JIC1 genome assembly compared with AL4, where the X chromosome is only the third longest scaffold and many additional genomic scaffolds with X-chromosome-like coverage patterns are unplaced (Li et al. 2019). This is likely due to improved resolution and representation of repetitive elements in JIC1 due to the use of long-read sequence data for de novode novo assembly. Indeed, for both *M. persicae* and *A. pisum*, we annotate a greater total length of repetitive DNA in our new assemblies than the previous versions that were based on short-read sequencing (*M. persicae* clone O: v1.1 = 57 Mb [16% of total assembly content], v2 = 88 Mb [22%]; *A. pisum*: Al4 = 154 Mb [29%], JIC1 = 178 Mb [34%]; supplementary fig. 6, Supplementary Material online).

### Extensive Autosomal Genome Rearrangement in Aphids

**Fig. 2.**
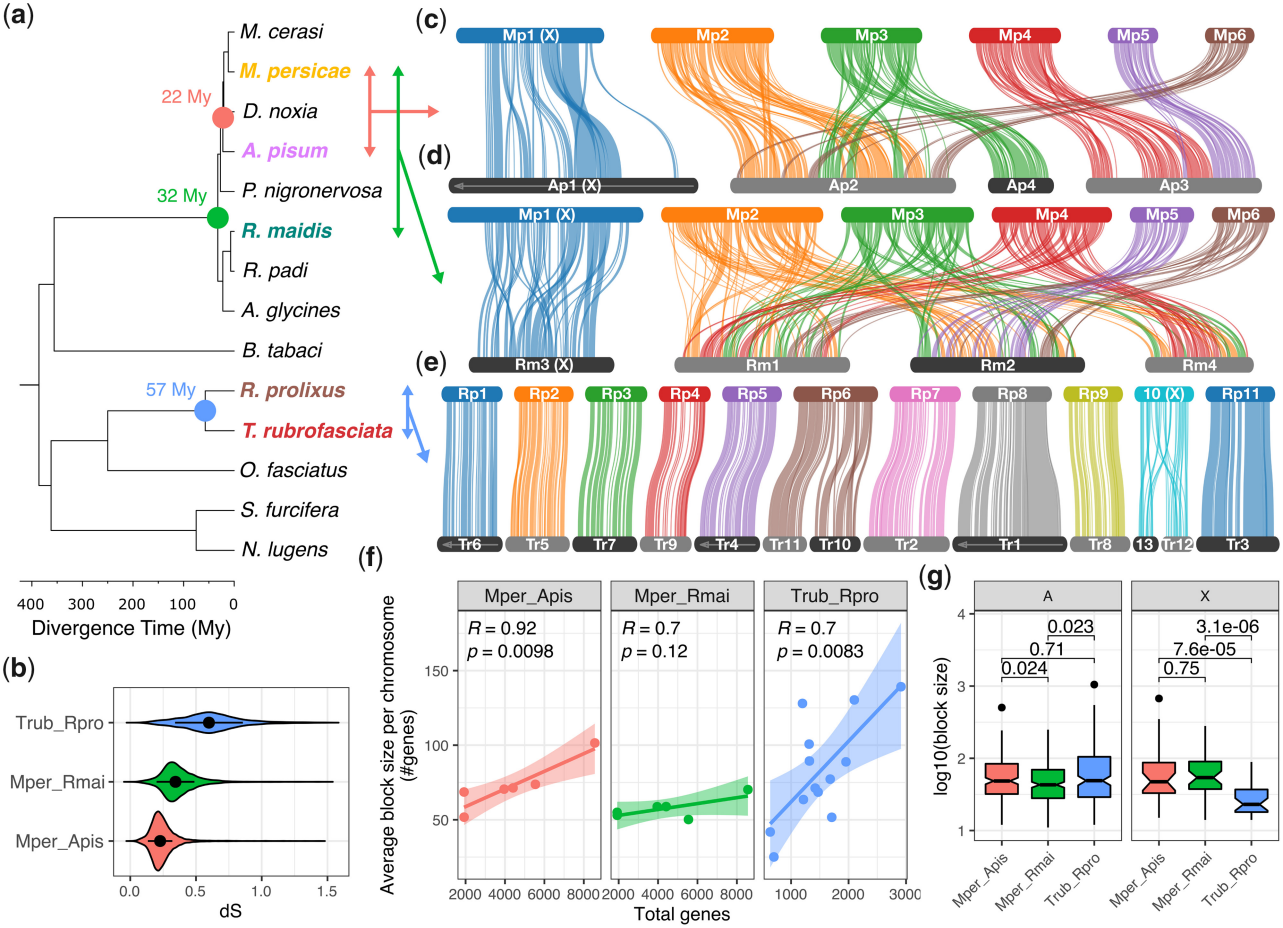
Divergent patterns of chromosome evolution across Hemiptera. (*a*) Time calibrated phylogeny of Hemiptera based on a concatenated alignment of 785 proteins conserved in all species. Divergence times were estimated using nonparametric rate smoothing with calibration nodes specified based on Johnson et al. (2018). Species with chromosome-scale genome assemblies are colored and divergence times between focal species are highlighted with colored circles. (*b*) Synonymous site divergence rate (d*S*) between *Triatoma rubrofasciata* and *Rhodnius prolixus* (blue), *Myzus persicae* and *Rhopalosiphum maidis* (green), and *M. persicae* and *Acyrthosiphon pisum* (red) based on 9,087, 7,965, and 9,290 syntenic one-to-one orthologs, respectively. Black circles and whiskers show median and interquartile range, respectively. (*c*–*e*) Pairwise synteny relationships within aphids (*c* and *d*) and Reduviidae (*e*) are mapped onto the phylogeny of Hemiptera. Links indicate the boundaries of syntenic gene blocks identified by MCScanX and are color coded by *M. persicae* (*c* and *d*) or *Rhod. prolixus* (*e*) chromosome ID. *Acyrthosiphon pisum* (*c*) and *R. maidis* (*d*) chromosomes are ordered based on *M. persicae*, and *T. rubrofasciata* (*e*) chromosomes are ordered according to *Rhod. prolixus*. Arrows along chromosomes indicate reverse compliment orientation relative to the focal species. Regions of chromosomes not joined by links lack detectable synteny at the resolution of our analysis. (*f*) The relationship between average synteny block size per chromosome (*y* axis) and chromosome size (*x* axis; measured as the total number of genes per chromosome). Trend lines show linear regression with 95% confidence intervals. For each comparison, the Pearson correlation coefficient (*R*) is given. (*g*) The size of MCScanX synteny blocks (measured in the number of genes within each block) located either on autosomes (A) or the X chromosome (X) for comparisons shown in (*c*)–(*e*). Numbers above comparisons show *P* values from Wilcoxon rank-sum tests.

To investigate aphid chromosome evolution, we identified syntenic genomic regions between *M. persicae*, *A. pisum*, and the published chromosome-scale assembly of *R. maidis* (Chen et al. 2019) using MCScanX (Wang et al. 2012), which identifies blocks of colinear genes (supplementary table 1, Supplementary Material online). *Myzus persicae* and *A. pisum* both belong to the aphid tribe Macrosiphini and diverged ∼22 Ma, whereas *R. maidis* belongs to Aphidini and diverged from *M. persicae* and *A. pisum* ∼33 Ma (fig. 2*a*). Assessment of chromosomal rearrangements shows a lack of large-scale rearrangements between the X chromosome and the autosomes for any of the aphid species analyzed, whereas aphid autosomes have undergone extensive structural change with many rearrangements between chromosomes (fig. 2*c* and *d*). Comparison between *M. persicae* and *A. pisum* within the tribe Macrosiphini reveals the signature of several chromosome fusion or fission events between autosomes that have occurred within the last 22 My (fig. 2*c*). For example, *M. persicae* scaffolds 4 and 5 are homologous to *A. pisum* scaffold 3, with the breakpoint clearly delineated. Comparing the more divergent species pair of *M. persicae* and *R. maidis*, which belong to Macrosiphini and Aphidini respectively, reveals highly rearranged autosomes with no clear homology (fig. 2*d*). This is also the case when comparing *R. maidis* to *A. pisum*, despite both species having the same 2*n* = 8 karyotype (supplementary fig. 7, Supplementary Material online), further supporting high levels of rearrangement. Similar results were obtained by mapping orthologs independently identified based on phylogenomic analysis of gene trees to *M. persicae* chromosomes (supplementary fig. 8 and table 2*a* and *b*, Supplementary Material online). In total, we identified 11,372 chromosomally placed one-to-one orthologs between *M. persicae* and *A. pisum* (41% of *M. persicae* genes) and 9,594 between *M. persicae* and *R. maidis* (35% of *M. persicae* genes). Using these data, we confirm that the aphid X chromosome is recalcitrant to translocations with the autosomes, with 93% (1,972/2,125) and 96% (1,388/1,452) of orthologs conserved on the X chromosome between *M. persicae* and *A. pisum* and between *M. persicae* and *R. maidis*, respectively. Taken together, our results show that the aphid X chromosome has been maintained for at least 33 My in contrast to extensive autosomal rearrangements.

### Divergent Patterns of Chromosome Evolution across Hemiptera

To investigate how aphid chromosome rearrangements compare with those of other hemipterans, we took advantage of two recently released chromosome-scale assemblies of the blood-feeding species *Rhod. prolixus* (obtained from the DNA Zoo; Dudchenko et al. 2017) and *T. rubrofasciata* (Liu et al. 2019). Both species belong to the hemipteran family Reduviidae and diverged from the aphid lineage ∼386 Ma (fig. 2*a*), representing a basal split in extant Hemiptera (Johnson et al. 2018). Unlike aphids, most Reduviidae have an XY chromosomal sex determination system (male = XY, female = XX) which is thought to be the ancestral state of Hemiptera (Blackmon et al. 2017) and reproduce exclusively through sexual reproduction. In some species, complex sex determination systems have been described with multiple X chromosomes (Ueshima 1966; Panzera et al. 1996). *Triatoma rubrofasciata* is one such species and has an X_1_X_2_Y male karyotype (Manna 1950). Multiple X chromosome systems in *Triatoma* are thought to be the result X chromosome fragmentation events (Ueshima 1966), we also examine this hypothesis here.

In striking contrast to aphids (fig. 2*c* and *d*), *Rhod. prolixus* and *T. rubrofasciata* have highly conserved synteny and an absence of translocation events between chromosomes (fig. 2*e*), despite being almost twice as divergent at the sequence level as the most divergent aphid comparison (fig. 2*b*; median synonymous site divergence: *M. persicae* vs. *R. maidis* = 34%, *T. rubrofasciata* vs. *Rhod. prolixus* = 60%). In total, just two chromosome fusion or fission events are detectable, one involving *Rhod. prolixus* chromosome 6 (Rp6) and a second involving the X chromosome (Rp10). The latter is likely an X chromosome fission in the *T. rubrofasciata* lineage which has led to the multiple X chromosome sex determination system observed in this species, supporting the hypothesis proposed by Ueshima over half a century ago (Ueshima 1966). For both the *M. persicae*–*A. pisum* comparison and the *T. rubrofasciata*–*Rhod. prolixus* comparison, synteny block size is positively correlated with chromosome length (fig. 2*f*). This relationship breaks down for the *M. persicae*–*R. maidis* comparison, again highlighting high rates of genome rearrangement in aphids. Indeed, despite higher sequence-level divergence, autosomal synteny blocks in Reduviidae are significantly larger than those identified between the most divergent aphid pair of *M. persicae* and *R. maidis* (Wilcoxon rank-sum test, *W* = 19,894, *P* = 0.02; fig. 2*g*) and are similar in size to those identified between the more closely related pair of *M. persicae* and *A. pisum* (Wilcoxon rank-sum test, *W* = 19,086, *P* = 0.71). This relationship is reversed for synteny blocks on the X chromosome which are significantly larger in aphids than Reduviidae (fig. 2*g*), whether comparing to *M. persicae*–*A. pisum* synteny blocks (Wilcoxon rank-sum test: *W* = 783, *P* = 7.55 × 10^−5^) or *M. persicae*–*R. maidis* synteny blocks (Wilcoxon rank-sum test: *W* = 1155, *P* = 3.08 × 10^−6^). Taken together, these results show divergent patterns of both inter- and intra-chromosomal rearrangement rates between aphids and Reduviidae, and that aphid diversification is associated with dynamic changes in autosome structure.

### TEs Are Enriched in Synteny Breakpoint Regions

**Fig. 3.**
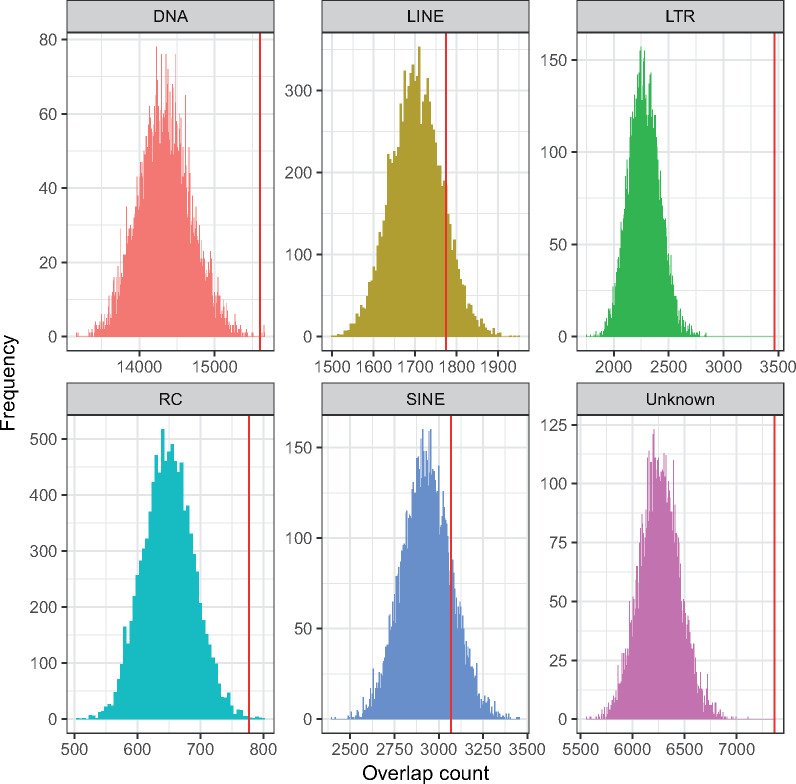
TEs are enriched within *Myzus persicae*–*Acyrthosiphon pisum* autosomal synteny breakpoint regions in the *M. persicae* clone O genome. *Histograms* show the distribution of TE counts (by class) in 10,000 randomized sets of autosomal regions with the same size distribution as observed *M. persicae*–*A. pisum* autosomal synteny breakpoint regions. Red lines indicate real observed values for each TE class within autosomal synteny breakpoint regions which shows that DNA transposons (DNA), long terminal repeat retrotransposons (LTR), rolling-circle Helitron transposons (RC), and unidentified transposons (Unknown) are significantly enriched in the breakpoint regions. The long and short interspersed nuclear elements (LINE and SINE, respectively) are not enriched.

Genome rearrangements may occur through nonallelic homologous recombination between repetitive elements (Mieczkowski et al. 2006; Chénais et al. 2012; Startek et al. 2015; Piazza and Heyer 2019). We hypothesized that repetitive elements are associated with the observed elevated rate of autosomal rearrangements in aphids. To test this, we compared TE content of autosomal synteny breakpoint regions (hereafter referred to as breakpoint regions) with those of conserved synteny blocks for the most recently diverged aphid species pair (i.e., *M. persicae* and *A. pisum*; fig. 2*c*). In total, breakpoint regions (excluding chromosome ends) span 34.5 Mb (12.4%) of autosomal sequence in *M. persicae* with an average length of 184 kb (*n* = 187, min = 60 bp, max = 2 Mb). TEs are highly enriched within breakpoint regions, accounting for 31.5% of all breakpoint region sequence compared with 17.9% in syntenic regions (supplementary table 3*a*, Supplementary Material online). TE content within breakpoint regions is nonrandom, with long terminal repeat (LTR) retrotransposons being most strongly enriched relative to random expectation (fig. 3 and supplementary table 3*a*, Supplementary Material online; permutation test: *P* < 0.0001). Indeed, despite representing only 12.4% of the genome, 29.5% of all autosomal LTR sequences are located within breakpoint regions, an enrichment of 2.38 times (supplementary table 3*a*, Supplementary Material online). Similar results were also found using the *A. pisum* JIC1 assembly as reference, with autosomal breakpoint regions strongly enriched for TEs compared with synteny blocks (44.6% vs. 28.1% TE content; supplementary table 3*b*, Supplementary Material online). As for *M. persicae*, the strongest enrichment of TEs within breakpoint regions was found for LTR elements (supplementary table 3*b* and fig. 9, Supplementary Material online; permutation test: *P* < 0.0001). Taken together, our results suggest that TE insertions may provide substrate for aphid genome rearrangement events.

### Conservation of Hemipteran X Chromosome Gene Content

**Fig. 4.**
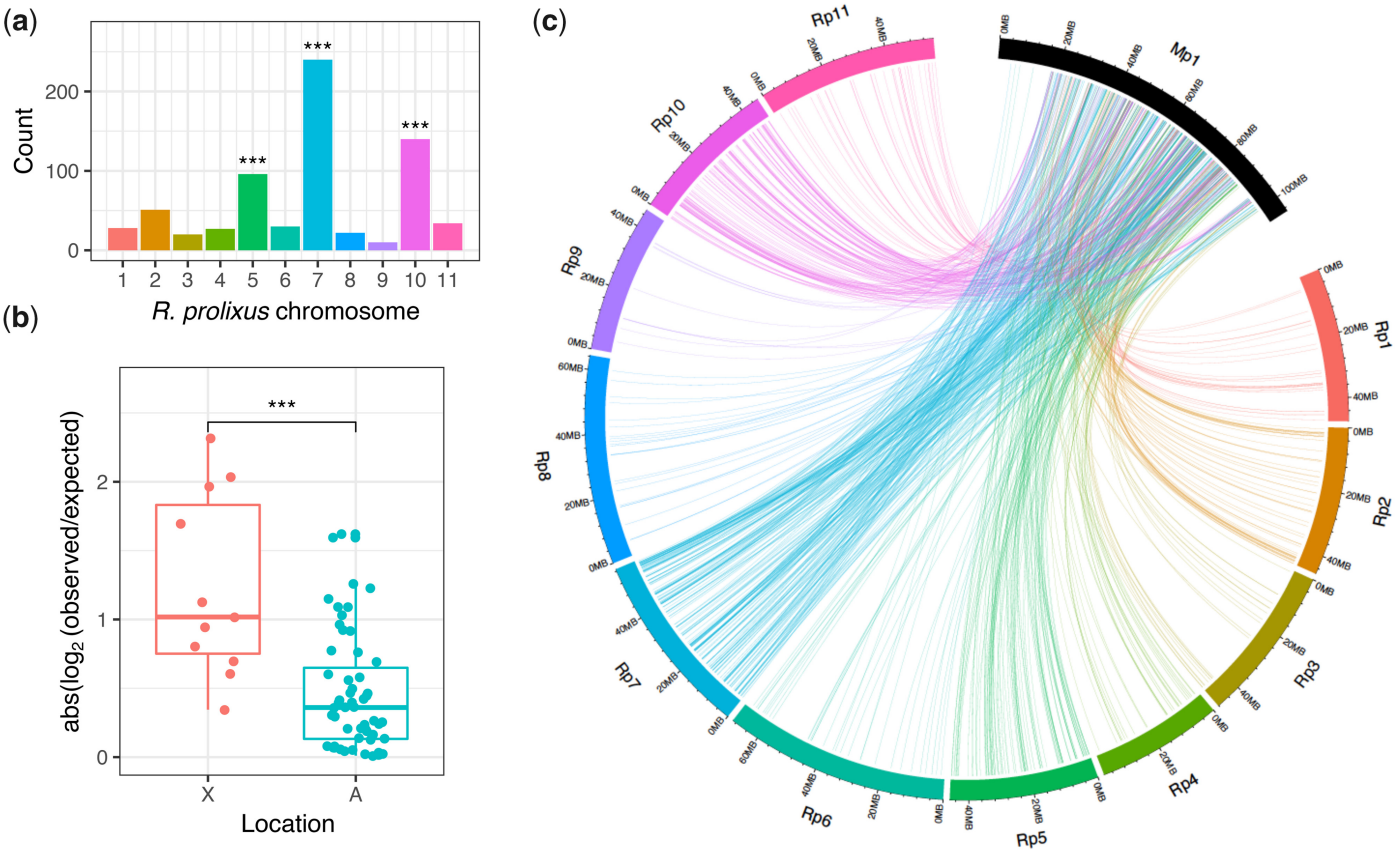
Ortholog mapping between the aphid *Myzus persicae* and the kissing bug *Rhodnius prolixus*. (*a*) Counts of *Rhod. prolixus* chromosomal location for 698 *M. persicae*–*Rhod. prolixus* 1:1 orthologs located on the *M. persicae* X chromosome (scaffold_1). Stars above bars indicate significant enrichment of a specific *Rhod. prolixus* chromosome after correcting for multiple testing (binomial test: BH corrected *P* < 0.05). (*b*) Absolute odds ratios (log_2_[observed/expected]) for *Rhod. prolixus* chromosomal enrichment on the *M. persicae* X chromosome and *M. persicae* autosomes. Each dot shows the odds ratio for a specific *Rhod. prolixus* chromosome. ***Wilcoxon rank-sum test *W* = 517, *P* = 2.31 × 10^−4^. (*c*) Chord diagram showing links between the *M. persicae* X chromosome (shown as Mp1) and the *Rhod. prolixus* chromosomes for 1:1 orthologs. Rp10 is the *Rhod. prolixus* X chromosome, the *Rhod. prolixus* Y chromosome is not assembled.

To test the hypothesis that the X chromosome is conserved across Hemiptera (Pal and Vicoso 2015), we compared our chromosome-scale assembly of *M. persicae* with *Rhod. prolixus*. We failed to identify syntenic blocks of genes between the two genome assemblies using MCScanX, probably due to the large evolutionary distance between *M. persicae* and *Rhod. prolixus* (386 My). Nonetheless, 6,191 one-to-one orthologs were identified between the two species (22% of *M. persicae* genes), 5,992 (97%) of which are anchored to chromosomes in both species. Using these orthologs, we find that the *M. persicae* X chromosome is significantly enriched for genes located on the *Rhod. prolixus* X chromosome (Rp10) (binomial test: Benjamini–Hochberg [BH] corrected *P* = 3.91 × 10^−13^; fig. 4*a* and *c* and supplementary fig. 10, Supplementary Material online), suggesting that the aphid and *Rhodnius* X chromosomes are homologous. Furthermore, absolute enrichment (and hence depletion) ratios of orthologs from specific *Rhod. prolixus* chromosomes were significantly higher for the *M. persicae* X chromosome than the autosomes (Wilcoxon rank-sum test: *W* = 517, *P* = 2.31 × 10^−4^; fig. 4*b* and supplementary table 4, Supplementary Material online), indicating that elevated conservation of the X chromosome, relative to autosomes, extends across Hemiptera. We also find that the *M. persicae* X chromosome is significantly enriched for genes that map to *Rhod. prolixus* autosomes Rp7 (binomial test: BH corrected *P* < 1.00 × 10^−16^) and Rp5 (binomial test: BH corrected *P* = 3.91 × 10^−13^) (fig. 4*a* and *c*). This suggests that the ancestral hemipteran X chromosome may have been fragmented in the *Rhod. prolixus* lineage or, alternatively, the aphid X chromosome may be a product of an ancient chromosome fusion event.

### The Aphid X Chromosome Is Repetitive, Depleted in Expressed Genes, and Rapidly Evolving

**Fig. 5.**
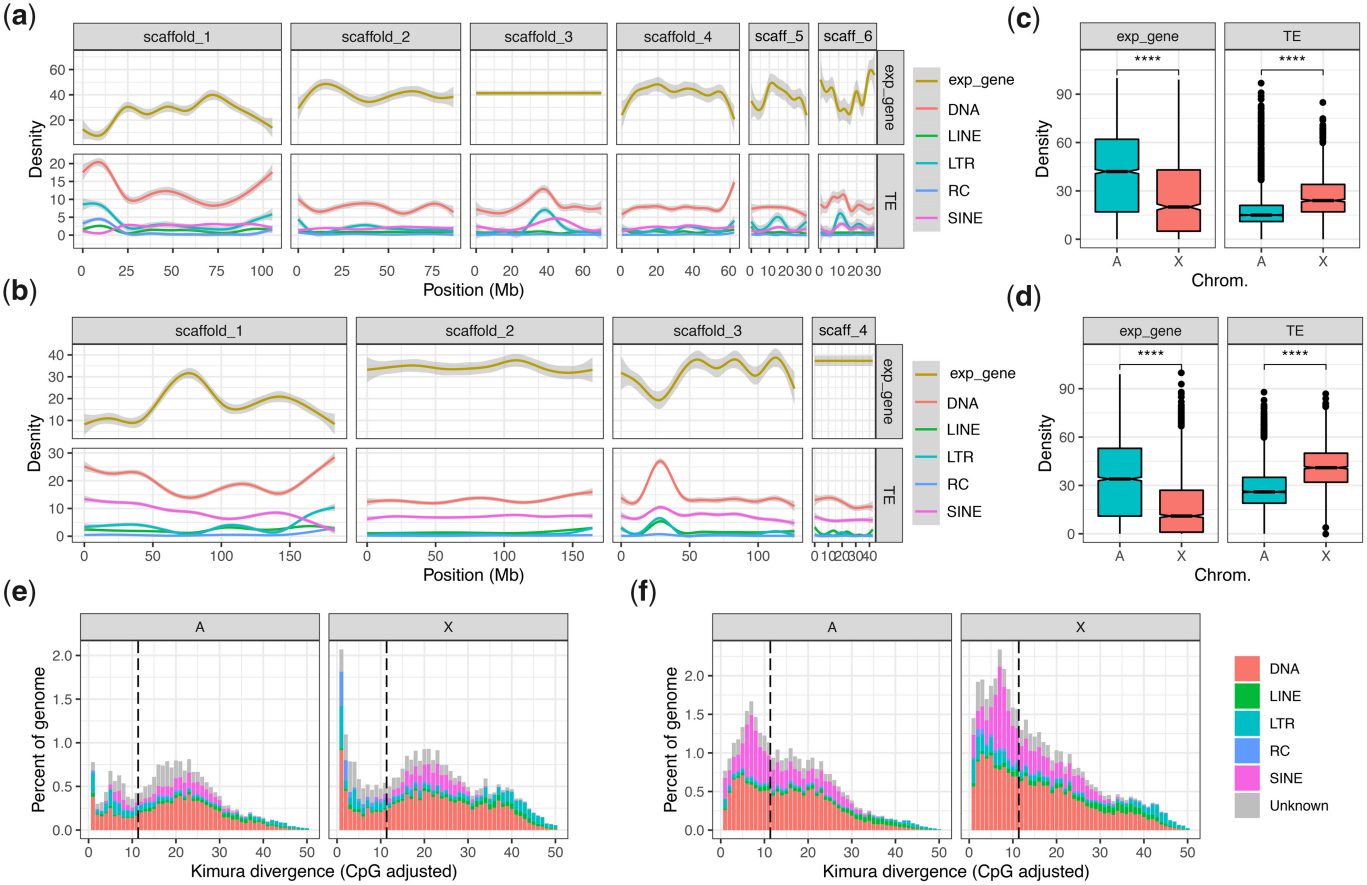
The aphid X chromosome is repetitive and depleted in expressed genes. (*a*) The density of expressed genes (expr_gene) and TEs across *Myzus persicae* clone O v2 chromosome-length scaffolds. Genes were classified as expressed if they had an estimated read count >4 in at least 12/24 *M. persicae* morph RNA-seq samples (see fig. 6). Lines show LOESS smoothed averages of 100-kb fixed windows. For detailed plots showing all data points for each feature class, see supplementary figures 14 and 15, Supplementary Material online. DNA, DNA transposons; LINE, long interspersed nuclear element; LTR, long terminal repeat retrotransposons; RC; rolling-circle transposons; SINE, short interspersed nuclear elements. (*b*) As for (*a*) but showing TEs and expressed genes across ApisJIC1 chromosome-length scaffolds. Genes were classified as expressed if they had an estimated read count >4 in at least 3/6 *Acyrthosiphon pisum* morph RNA-seq samples from Jaquiéry et al. (2013). (*c*) *Box plots* showing median density of expressed genes and TEs in 100-kb fixed windows across *M. persicae* autosomes and the X chromosome. The X chromosome has significantly lower gene density (Wilcoxon rank-sum test: *W* = 1,934,963, *P* < 2.2 × 10^−16^) and significantly higher TE density (Wilcoxon rank-sum test: *W* = 786,210, *P* < 2.2 × 10^−16^) than the autosomes. (*d*) *Box plots* showing median density of expressed genes and TEs in 100-kb fixed windows across *A. pisum* clone JIC1 autosomes and the X chromosome. The X chromosome has significantly lower gene density (Wilcoxon rank-sum test: *W* = 16,062,992, *P* < 2.2 × 10^−16^) and significantly higher TE density (Wilcoxon rank-sum test: *W* = 6,340,780, *P* < 2.2 × 10^−16^) than the autosomes. (*e*) *Stacked histograms* showing the age distribution of TEs located on *M. persicae* clone O autosomes (A) and the X chromosome (X). TE families are grouped as for (*a*) and (*b*). The dashed black line indicates half the median synonymous site divergence (11.35%) between *M. persicae* and *A. pisum* one-to-one orthologs and is a proxy for the divergence time, that is, TE insertions with lower divergence from their respective consensus sequence than this point likely arose after *M. persicae* and *A. pisum* diverged. (*f*) As for (*e*) but for *A. pisum* clone JIC1.

Conservation of aphid X chromosome gene content is remarkable given its dynamic genomic substrate. In *M. persicae* and *A. pisum*, the X chromosome is significantly more repetitive than the autosomes and significantly depleted in expressed genes (fig. 5*a*–*d*). Across the *M. persicae* X chromosome, 27% of bases are annotated as TEs compared with 19% in autosomes (*χ*^2^ = 3,486,014, df = 1, *P *<* *2.2 × 10^−16^). The *A. pisum* X chromosome is even more repetitive, with 42% of bases annotated as TEs compared with 29% in autosomes (*χ*^2^ = 8,455,518, df = 1, *P *<* *2.2 × 10^−16^). The ends of the X chromosome in both *M. persicae* and *A. pisum* appear to be gene expression deserts with low numbers of expressed genes relative to the autosomes and to the central regions of the X chromosome (fig. 5*a* and *b*). These gene-poor regions have significant reduction in the density of expressed genes toward the telomeres (*M. persicae*: Pearson correlation [*R*] = −0.46, *P* = 6.4 × 10^−7^; *A. pisum*: *R* = −0.46, *P* = 5.1 × 10^−11^; supplementary figs. 11 and 12, Supplementary Material online). This reduction is associated with significant increases in the densities of DNA transposons (*M. persicae*: *R *=* *0.51, *P* = 1.9 × 10^−8^; *A. pisum*: *R *=* *0.63, *P* < 2.2 × 10^−16^), LTR retrotransposons (*M. persicae*: *R *=* *0.52, *P* = 1.0 × 10^−8^; *A. pisum*: *R *=* *0.46, *P* = 4.4 × 10^−11^), and rolling-circle Helitron transposons (*M. persicae*: *R *=* *0.50, *P* = 6.5 × 10^−8^; *A. pisum*: *R *=* *0.38, *P* = 1.2 × 10^−7^) (fig. 5*a* and *b* and supplementary figs. 11 and 12, Supplementary Material online). There is also a weak but significant increase in long interspersed nuclear elements (LINE) toward the ends of the X chromosome in both species (*M. persicae*: *R *=* *0.20, *P* = 0.04; *A. pisum*: *R *=* *0.16, *P* = 0.029).

The invasion of the aphid X chromosome by TEs appears to be ongoing, with many young TEs annotated in both *M. persicae* and *A. pisum* (fig. 5*e* and *f*). This is particularly pronounced in *A. pisum* where X chromosome TE dynamics have had a substantial influence on the size of the *A. pisum* genome. Overall, the *A. pisum* JIC1 assembly is 131 Mb (33%) larger than the *M. persicae* clone O v2 assembly (table 1; 526 vs. 395 Mb). Strikingly, 59% of this difference is due to the size of the X chromosome, which is 78 Mb larger (74%) in *A. pisum* (X chromosome = 183 Mb) than *M. persicae* (X chromosome = 105 Mb). Given we can rule out X chromosome–autosome fusions in *A. pisum* based on our synteny analysis (fig. 1*c*), the difference in X chromosome size is the product of expansion in *A. pisum* and/or contraction in *M. persicae*. Although both of these factors likely play a role, our analysis of *A. pisum* TE dynamics indicates that lineage-specific TE expansion in *A. pisum* accounts for a substantial proportion of the observed size difference compared with *M. persicae*. We base this conclusion on the relatively young age of the TEs in the X chromosome of *A. pisum*. Using the conservative estimate that the substitution rate of TE insertions is equivalent to that of synonymous sites in protein-coding genes (i.e., approximately neutral), the *A. pisum* X chromosome contains 41 Mb of TE insertions that likely accumulated since *A. pisum* and *M. persicae* diverged (fig. 5*f*; divergence from consensus <11.35%). In other words, recent TE insertions on the *A. pisum* X chromosome account for ∼53% of the X chromosome size difference compared with *M. persicae*.

As well as being repetitive, we also find that genes on the *M. persicae* X chromosome have a higher rate of evolution (measured using the ratio of the nonsynonymous to synonymous nucleotide substitutions) than those on the autosomes (supplementary fig. 13 and table 1, Supplementary Material online), a phenomenon previously observed in *A. pisum* (Jaquiéry et al. 2012, 2018). Our results are therefore consistent with a “fast-X” effect operating across aphids. Stability of aphid X chromosome gene content has therefore been maintained in the face of extensive historical, and ongoing, TE activity and high rates of sequence evolution.

#### Patterns of Gene Expression along the M. persicae Genome

**Fig. 6.**
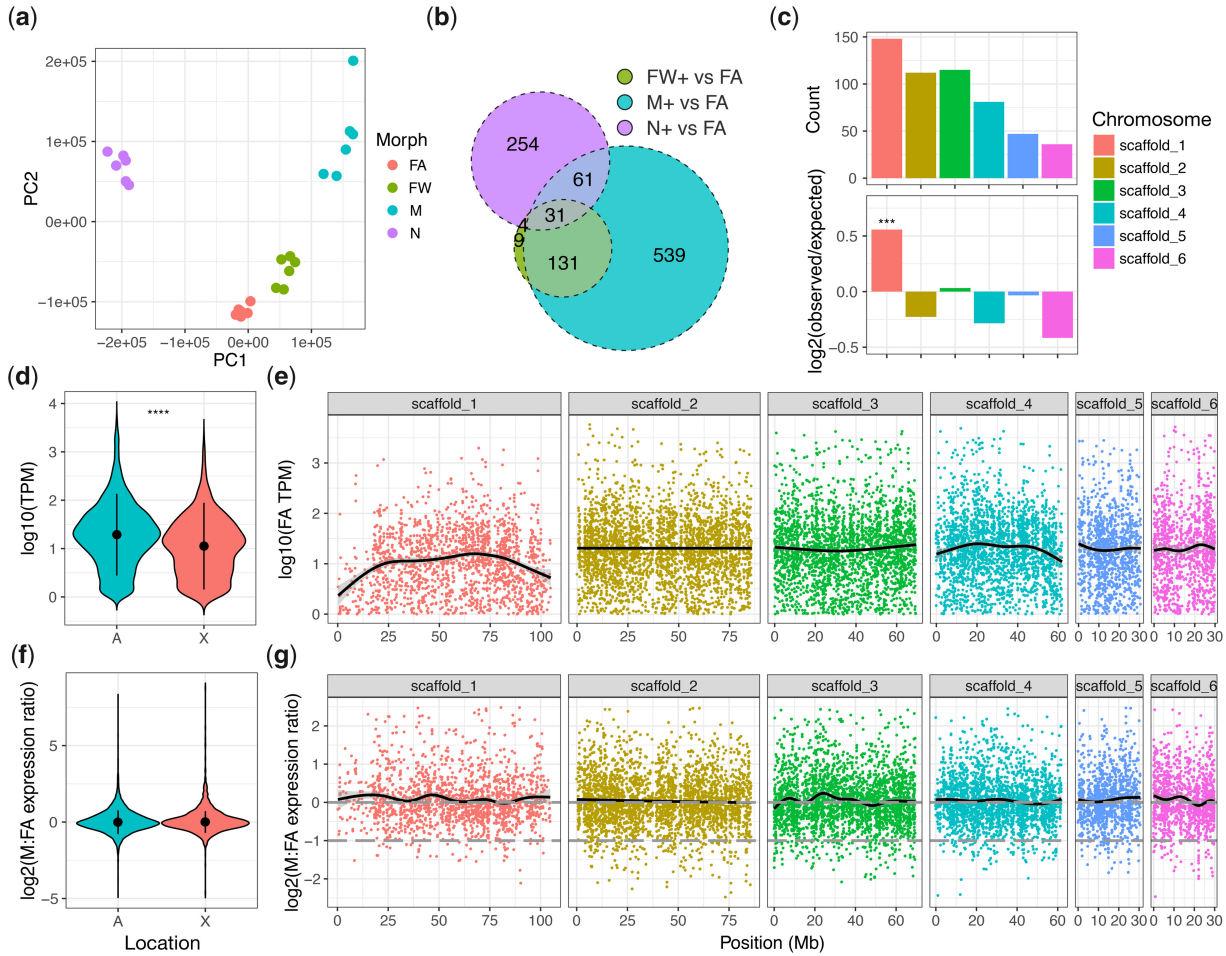
Patterns of gene expression in *Myzus persicae* morphs and along *M. persicae* clone O v2 chromosome-length scaffolds. (*a*) Principle component analysis (PCA) based on RNA-seq gene expression levels in whole bodies of *M. persicae* clone O unwinged asexual females (FA), winged asexual females (FW), winged males (M), and nymphs (N). Each morph has a distinct gene expression profile with tight clustering of replicates (*n* = 6 per morph). (*b*) Overlap of genes upregulated in either M, FW, or N relative to FA (Sleuth likelihood ratio test: *q* < 0.05, effect size (beta) > 0.5). (*c*) The distribution of genes specifically upregulated in males (*n* = 539) across *M. persicae* clone O v2 chromosome-length scaffolds. Top panel shows counts of M-biased genes per scaffold. Bottom panel shows enrichment scores (log_2_[observed/expected]) of M-biased genes per scaffold relative to the total number of expressed genes on each scaffold (estimated read count >4 in at least 12/24 RNA-seq samples). Significant enrichment was assessed using a binomial test (*P* < 0.05) with the number of trials equal to the count of expressed genes per scaffold and the probability of success equal to the overall proportion of M-biased genes located on chromosomes relative to the number of expressed genes on all chromosomes. Only the X chromosome is significantly enriched for M-biased genes. ***Binomial test: *P* = 2.38 × 10^−6^. (*d*) *Violin plots* showing the distribution of log_10_ average expression levels (measured in TPM) in FA of expressed genes (TPM > 1) located on *M. persicae* autosomes (A) and the X chromosome (X). The X chromosome has significantly lower gene expression levels than the autosomes (Wilcoxon rank-sum test: *W* = 715,820, *P* < 2.2 × 10^−16^). (*e*) FA gene expression ratios used in (*d*) across *M. persicae* clone O v2 chromosome-length scaffolds. Each dot corresponds to a gene, the black line shows the LOESS smoothed average. (*f*) *Violin plots* showing the distribution of log_2_ M to FA gene expression ratios on *M. persicae* autosomes (A) and the X chromosome (X) for genes with average expression of at least 1 TPM in M and FA. Black circles and lines within the colored regions indicate the median an interquartile range, respectively. There is no significant difference between A and X (Wilcoxon rank-sum test: *W* = 8,919,400, *P* = 0.10). (*g*) The distribution of log_2_ M to FA gene expression ratios used in (*f*) across *M. persicae* clone O v2 chromosome-length scaffolds. Each dot corresponds to a gene, the black line shows the LOESS smoothed average. The dashed gray lines indicate the expected M to FA gene expression ratio given full dosage compensation (log_2_[M:FA] expression = 0) and in the absence of dosage compensation (log_2_[M:FA] expression = 0.5). Extremely M-biased or FA-biased genes (abs. log_2_ M:FA expression ratio > 2.5) are excluded.

Unlike other systems where a fast-X effect is observed (Mank et al. 2010), rapid evolution of the aphid X chromosome cannot be explained by reduced efficacy of selection caused by a lower effective population size of the X chromosome relative to autosomes (Jaquiéry et al. 2012). This is because progeny produced by aphid sexual reproduction are exclusively female (XX) and inherit an X chromosome from both of their parents, leading to an equivalency of effective population size between the X chromosome and the autosomes (Jaquiéry et al. 2012). Rather, aphid fast-X evolution is thought to be predominantly explained by patterns of gene expression. Specifically, lower gene expression levels of X-linked genes compared with those on the autosomes, and enrichment of genes expressed in rare morphs, that is, males and sexual females, possibly driven by antagonistic selection (Jaquiéry et al. 2018). Both of these factors lead to relaxed purifying selection on X-linked genes. We examined these hypotheses using our new chromosome-scale assembly of *M. persicae* and a large gene expression data set for diverse *M. persicae* morphs. In particular, we investigated genome-wide patterns of gene expression in unwinged asexual females, winged asexual females, winged males, and unwinged asexual female nymphs (fig. 6*a*). We identified 5,046 differentially expressed genes between *M. persicae* morphs assuming a 5% false discovery rate (Sleuth likelihood ratio test: *q* < 0.05, absolute effect size [beta] > 0.5 relative to asexual female morphs; supplementary table 5, Supplementary Material online). Out of a total of 1,029 morph-biased genes, 539 (52.4%) are specifically upregulated in males relative to the common wingless asexual female morph (fig. 6*b*). These male-biased genes are significantly enriched on the *M. persicae* X chromosome (binomial test: *P* = 2.38 × 10^−6^; fig. 6*c*), confirming our previous results obtained using a fragmented genome assembly (Mathers et al. 2019) and matching patterns of male-biased gene expression observed in *A. pisum* (Jaquiéry et al. 2013; Purandare et al. 2014; Pal and Vicoso 2015). Using gene expression data for asexual females, we confirm that the X chromosome has significantly lower gene expression than the autosomes (Wilcoxon rank-sum test: *W* = 715,820, *P* < 2.2 × 10^−16^; fig. 6*d*) and that this is particularly pronounced for the 5′ and 3′ ends of the chromosome (fig. 6*e*).

Finally, we also confirm the operation of dosage compensation in *M. persicae*; despite the X chromosome being found as a single copy in males, there was no significant difference observed in the male to asexual female gene expression ratio between the X chromosome and the autosomes (Wilcoxon rank-sum test: *W* = 8,919,400, *P* = 0.10; fig. 6*f* and supplementary table 6, Supplementary Material online). Dosage compensation has previously been shown to operate in other Hemiptera (Pal and Vicoso 2015) and in *A. pisum* (Jaquiéry et al. 2013; Richard et al. 2017) using fragmented assemblies. Using our new chromosome-scale assembly of *M. persicae*, we are able to show that dosage compensation operates across the entire X chromosome (fig. 6*g*).

## Conclusion

We find that three aphid species within the subfamily Aphidinae, that span ∼30 My of aphid evolution, show extensive autosomal genome rearrangements. This is in contrast to other insect genomes that have been compared thus far, including within Lepidoptera and Diptera. Furthermore, the high rate of autosomal rearrangements does not appear to be a ubiquitous feature of Hemiptera given that two other Hemiptera (*Rhod. prolixus* and *T. rubrofasciata*) have highly conserved synteny (fig. 2*e*). Our data support previous karyotype studies showing that chromosome numbers are highly variable among aphids (Blackman 1980). Furthermore, our data reveal that aphid chromosome number variation is not only caused by chromosome fission or fusion (i.e., macromutations) but also caused by interautosomal translocation events. In contrast to the autosomes, the aphid X chromosome appears to recalcitrant to rearrangement with the autosomes, and it appears structurally highly conserved. The long-term stability of aphid X chromosome gene content is surprising, given that we observed low levels of gene expression of X-linked genes, relaxed selection on coding genes, and an accumulation of TEs. This implies that strong selection may be acting against interchromosomal rearrangements involving the X chromosome in aphids. It is possible that large-scale translocations involving the X chromosome interfere with dosage compensation, causing the misexpression of genes (Sharp et al. 2002). Alternatively, intact X chromosomes may be required for proper elimination of the X chromosome during male determination. If X chromosome conservation is not caused by natural selection, there might be an as yet unidentified process that curbs the rate of rearrangement of this chromosome.

A recent study by Li et al. (2020), published shortly after the early release of our results (Mathers et al. 2020b), also revealed high rates of autosomal genome rearrangement in aphids and conservation of the X chromosome. Li et al. (2020) compared a chromosome-scale assembly of *A. pisum* (AL4; Li et al. 2019) with the published assembly of *R. maidis* (Chen et al. 2019). Here, we generated another chromosome-scale assembly of a different *A. pisum* isolate (JIC1) using long-read sequencing, linked-read sequencing, and chromatin conformation capture (HiC). Compared with AL4, the assembly of JIC1 is more contiguous, allowing better comparison among aphid species. In particular, by using long-read sequencing, we dramatically improve the assembly of the *A. pisum* X chromosome, incorporating an additional 50 Mb of sequence. Moreover, this study included a highly contiguous chromosome-level assembly of another aphid species, *M. persicae*, which belongs to a different clade within Macrosiphini, whereas *R. maidis* belongs to the tribe Aphidini. By including more closely related aphid species, we demonstrate that the high rate of autosomal rearrangement in aphids appears to be ongoing, at least within Aphidinae (Macrosiphini + Aphidni).

Li et al. (2020) also confirm previously described features of pea aphid gene expression and genome architecture (Jaquiéry et al. 2013; Purandare et al. 2014; Richard et al. 2017), showing that the X chromosome has lower gene expression levels than the autosomes, that dosage compensation operates on X-linked genes and that the X chromosome is enriched in genes with male-biased expression. We confirm the generality of these findings using our new high-quality genome assembly of *M. persicae* and a comprehensive transcriptomic data set of diverse *M. persicae* morphs.

With the improved long-read genome assembly of *A. pisum* and the high-quality long-read assembly of *M. persicae* in hand, we were able to carry out a detailed analysis of repeat evolution in aphids, gaining insights into both X chromosome and autosome evolution. We find that the large difference in genome size observed between *M. persicae* and *A. pisum* has been substantially influenced by recent TE activity on the *A. pisum* X chromosome. We also find evidence that repeats may be playing an important role in the high rate of genome rearrangement observed in aphids with significant enrichment of LTR retrotransposons, DNA transposons, and rolling-circle Helitron transposons found within synteny breakpoint regions.

Li et al. (2020) compared *A. pisum* (AL4) and *R. maidis* with a chromosome-level genome assembly of a psyllid (*Pachypsylla venusta*), which, like aphids, belongs to the suborder Sternorrhyncha. This revealed low levels of synteny and distinct patterns of sex-biased gene expression and selection on the psyllid X chromosome compared with the aphid X chromosome. We extend the analysis of hemipteran chromosome evolution across the full span of the order by including two blood-feeding members of Reduviidae (Hemiptera: Heteroptera), which represent a basal split within Hemiptera relative to aphids (fig. 2*a*). The inclusion of these additional species reveals a surprising divergence in hemipteran autosome evolution, with high synteny observed between the two investigated Reduviidae species contrasting with extensive rearrangement in aphids. This is a significant observation as it suggests that the presence of holocentric chromosomes alone does not explain the observed high rate of autosomal genome rearrangement in aphids given that holocentricity is conserved across Hemipetra (Melters et al. 2012). Additionally, by including a comparison across Hemiptera, we are able to confirm the hypothesis of Pal and Vicoso (2015) that the hemipteran X chromosome has substantial conservation of gene content.

Altogether, this study shows that long-read sequencing and chromosome-scale assemblies can uncover large-scale rearrangement events that are likely to have significantly impacted aphid genome evolution. We show that repeats are likely to play an important role in driving genome rearrangements in aphids. As such, aphids serve as an excellent model system to understand the role of genome rearrangements in species radiations and adaptation.

## Materials and Methods

### Aphid Genome Assembly Strategy

To assemble high-quality reference genomes for *M. persicae* and *A. pisum*, we generated initial de novode novo contig assemblies based on high-coverage Nanopore long-read data. These assemblies were then scaffolded into pseudomolecules (chromosomes) using in vivoin vivo chromatin conformation capture (HiC) data (Dudchenko et al. 2017) and, in the case of *A. pisum*, 10X Genomics Chromium linked reads (Zheng et al. 2016; Weisenfeld et al. 2017). As *M. persicae* and *A. pisum* have divergent genome architectures (e.g., repeat content and level of heterozygosity), we optimized the initial contig assembly for each species, aiming to maximize genome completeness and minimize pseudo duplication caused by undercollapsed heterozygosity. These criteria were assessed by comparing the K-mer content of raw sequencing reads to the genome assembly with the K-mer Analysis Toolkit (Mapleson et al. 2017) and by assessing the representation of conserved genes with BUSCO v3 (Simão et al. 2015; Waterhouse et al. 2018), using the Arthropoda gene set (*n* = 1,066). We also used genome size estimates for *M. persicae* (409 Mb) and *A. pisum* (514 Mb) based on flow cytometry from Wenger et al. (2020) to assess the proportion of the genome that had been assembled and to estimate sequence read coverage. For each species, we compared long-read assemblies generated with Canu (Koren et al. 2017), Flye (Kolmogorov et al. 2019), and wtdbg2 (Ruan and Li 2019) as well as various combinations of assembly merging with quickmerge (Chakraborty et al. 2016), the effect of removing alternative haplotypes and the effect of long- and short-read assembly polishing (supplementary note, Supplementary Material online). Below, we describe the steps used to generate the final genome assembly for each species.

### Sequencing and De Novo Assembly of *M. persicae* Clone O

We previously sequenced the genome of *M. persicae* clone O using Illumina short-read sequencing (Mathers et al. 2017). We used aphids derived from the same asexually reproducing colony maintained at the John Innes Centre insectary for all DNA extractions.

For Nanopore long-read sequencing, batches of 20 aphids were collected in 1.5-ml low-bind Eppendorf tubes and snap frozen in liquid nitrogen. We extracted high molecular weight DNA with the Illustra Nucleon PhytoPure kit (GE Healthcare, RPN8511) following the manufacturers protocol. Wide-bore pipette tips were used when transferring solutions to circumvent shearing of DNA. DNA concentration was determined using the Qubit broad-range assay. The purity of each extraction was assessed using a NanoDrop spectrophotometer (Thermo Fisher) based on 260/280 and 260/230 nm absorbance values, and by comparing the NanoDrop concentration estimate to the Qubit estimate, looking for a ratio close to 1:1 (Schalamun et al. 2019). The length of extracted DNA molecules was assessed using a Femto fragment analyzer (Agilent). Nanopore genomic DNA libraries were prepared for samples passing quality control using the Ligation Sequencing Kit (Oxford Nanopore Technologies [ONT], Oxford, UK: SQK-LSK109) following the manufacturers protocol with the exception that we started with 10 µg of high molecular weight DNA. In total, four libraries were generated and each one sequenced on an R9.4 flow cell for 72 h. Base calling was run using Guppy v2.3.1 (ONT, Oxford, UK) with default settings, retaining reads with a quality score of at least 7. This resulted in a total of 28 Gb of data (∼70× coverage of the *M. persicae* genome) with a an N50 of 23 kb (supplementary table 7, Supplementary Material online).

We also generated 24 Gb (∼59× coverage) of Illumina short reads for assembly polishing and quality control. DNA was extracted from ∼50 individuals with a modified CTAB protocol (Marzachi et al. 1998) and sent to Novogene (China) for sequencing. Novogene prepared a polymerase chain reaction-free Illumina sequencing library using the NEBNext Ultra II DNA Library Prep Kit for Illumina (New England Biolabs, USA), with the manufacturers protocol modified to give a 500-bp–1-kb insert size. This library was sequenced on an Illumina HiSeq 2500 instrument with 250-bp paired-end chemistry. The resulting reads were trimmed for adapter sequences with trim_galore! v0.4.0 (Krueger 2015), retaining read pairs where both sequences were at least 150-bp long after adapter trimming.

In our exploratory analysis, wtdgb2 v2.3 gave optimum performance for assembling the *M. persicae* clone O Nanopore data (supplementary note, Supplementary Material online). We generated two wtdgb2 assemblies with the parameters “-x ont -p 0 -k 17 -L 15000” and “-x ont -p 19 -k 0 -L 15000.” These assemblies had complementary contiguity and contained nonoverlapping sets of BUSCO genes. We therefore merged the two wtdgb2 genome assemblies with quickmerge v0.3 using the parameters “-l 1837291 -ml 10000,” with the more complete wtdgb2 “-x ont -p 0 -k 17 -L 15000” assembly used as the query. This resulted in an assembly that was more complete and more contiguous than either individual wtdgb2 assembly (see supplementary note, Supplementary Material online). The merged wtdgb2 assembly was then iteratively polished, first with three rounds of long-read polishing with racon v1.3.1 (Vaser et al. 2017), then with three rounds of short-read polishing with Pilon v1.22 (Walker et al. 2014) in diploid mode. Redundant haplotigs (contigs derived from uncollapsed heterozygosity) were removed from the polished assembly with Purge Haplotigs (Roach et al. 2018) using the sequence coverage bounds 9, 45, and 92, and requiring contigs to cover at least 90% of another, longer contig, to be flagged as a haplotig.

### Sequencing and De Novo Assembly of *A. pisum* Clone JIC1

An isolate of *A. pisum* (dubbed JIC1) found on *Lathyrus odoratus* (sweet pea) was collected from Norwich in 2005 and subsequently reared at the JIC insectary under controlled conditions (Bedford I, personal communication). DNA extractions and Nanopore sequencing libraries were prepared as described above for *M. persicae* clone O. In total, two libraries were generated and each one sequenced on an R9.4 flow cell for 72 h. Base calling was run using Guppy v2.3.1 with the “flip-flop” model, retaining reads with a quality score of at least 7. This resulted in a total of 18 Gb of data (∼35× coverage of the *A. pisum* genome) with an N50 of 33 kb (supplementary table 7, Supplementary Material online).

To improve the Nanopore de novo assembly and generate accurate Illumina short reads for assembly polishing, we generated 10X Genomics Chromium linked-read data using DNA extracted as described above. High molecular weight DNA was sent to Novogene (China) for 10X Genomics Chromium library preparation following the manufacturers protocol and sequencing was performed on an Illumina NovaSeq instrument. In total we generated 45 Gb of 150-bp paired-end reads (∼88× coverage of the *A. pisum* genome). The average molecule size of the library was 32 kb (supplementary table 7, Supplementary Material online).

De novo assembly with Flye v2.4 using default settings gave the best balance between contiguity, genome completeness, and absence of erroneously duplicated content (supplementary note, Supplementary Material online). The Flye assembly was polished as described above for *M. persicae*, with three rounds of racon followed by three rounds of Pilon. For Pilon polishing, we used the 10X reads after removing barcodes and primer sequence with process_10xReads.py (https://github.com/ucdavis-bioinformatics/proc10xG, last accessed March 25, 2020). Redundant haplotigs were removed from the polished Flye assembly with Purge Haplotigs (Roach et al. 2018) using the sequence coverage bounds 4, 21, and 57, and requiring contigs to cover at least 75% of another, longer contig, to be flagged as a haplotig. Finally, we iteratively scaffolded the deduplicated Flye assembly using our 10X Genomics linked-read data. We ran two iterations of Scaff10x v4.0 (https://github.com/wtsi-hpag/Scaff10X, last accessed March 25, 2020) with the parameters “-longread 1 -edge 45000 -block 45000” followed by Tigmint v1.1.2 (Jackman et al. 2018) with default settings, which identifies misassemblies, breaks the assembly, and performs a final round of scaffolding with ARCS (Yeo et al. 2018).

### HiC Libraries and Genome Scaffolding

To scaffold our de novo assemblies of *M. persicae* clone O and *A. pisum* clone JIC1, we used in vivoin vivo chromatin conformation capture to generate HiC data. For each species, whole bodies of individuals from the same clonal populations used for genome sequencing were snap frozen in liquid nitrogen and sent to Dovetail Genomics (Santa Cruz, CA) for HiC library preparation and sequencing. HiC libraries were prepared using the *Dpn*II restriction enzyme following a similar protocol to Lieberman-Aiden et al. (2009). HiC libraries were sequenced on an Illumina HiSeq X instrument, generating 150-bp paired-end reads. In total, we generated 123 Gb (∼300× coverage) and 21 Gb (∼40× coverage) of HiC data for *M. persicae* clone O and *A. pisum* clone JIC1, respectively (supplementary table 7, Supplementary Material online). To identify HiC contacts, we aligned our HiC data to our draft assemblies using the Juicer pipeline (Durand et al. 2016). We then used the 3D-DNA assembly pipeline (Dudchenko et al. 2017) to first correct misassemblies in each input assembly and then to order contigs (or scaffolds for *A. pisum* JIC1) into superscaffolds. K-mer analysis showed that our draft assemblies did not contain substantial quantities of duplicated content caused by the inclusion of haplotigs so we ran 3D-DNA in “haploid mode” with default settings for *M. persicae* clone O and “--editor-repeat-coverage 4” for *A. pisum* JIC1 (supplementary note, Supplementary Material online). The initial HiC assembly for each species was then manually reviewed using Juicebox Assembly Tools to correct misjoins and other errors (Dudchenko et al. 2018). Following Juicebox Assembly Tools review, the assemblies were polished with the 3D-DNA seal module to reintegrate genomic content removed from superscaffolds by false positive manual edits to create a final scaffolded assembly. The HiC assemblies were then screened for contamination with BlobTools (Kumar et al. 2013; Laetsch and Blaxter 2017). Finally, a frozen release was generated for each assembly with scaffolds renamed and ordered by size with SeqKit v0.9.1 (Shen et al. 2016). The final assemblies were checked with BUSCO and K-mer Analysis Toolkit comp to ensure the scaffolding and decontamination steps had not reduced gene-level completeness or removed genuine single-copy aphid genome content.

### Transcriptome Sequencing of *M. persicae* Morphs

We previously sequenced the transcriptomes *M. persicae* clone O apterous (unwinged) asexual females and alate (winged) males using six biological replicates per morph (Mathers et al. 2019). As part of the same experiment, we also collected and sequenced nymphs (derived from apterous asexual females) and alate asexual females (also six biological replicates each). These data were not used in our original study (Mathers et al. 2019) but are included here for genome annotation and to provide a more comprehensive view of morph-biased gene expression in *M. persicae*. Aphid rearing, RNA extraction, and sequencing were carried out as in Mathers et al. (2019). Apterous asexual females, alate asexual females, and nymphs were reared in long day conditions (14 h light, 22 °C day time, and 20 °C night time, 48% relative humidity) and alate males were reared in short day conditions (8 h light, 18 °C day time, and 16 °C night time, 48% relative humidity).

### Genome Annotation

We annotated protein-coding genes in our new chromosome-level assemblies of *M. persicae* and *A. pisum* using BRAKER2 v2.1.2 (Hoff et al. 2016, 2019), incorporating evidence from RNA-seq alignments. Prior to running BRAKER2, we soft-masked each genome with RepeatMasker v4.0.7 (Tarailo-Graovac and Chen 2009; Smit et al. 2015) using known Insecta repeats from Repbase (Bao et al. 2015) with the parameters “-e ncbi -species insecta -a -xsmall -gff.” We then aligned RNA-seq data to the soft-masked genomes with HISAT2 v2.0.5 (Kim et al. 2015). All RNA-seq data sets used for annotation are summarized in supplementary table 8, Supplementary Material online. For *M. persicae*, we aligned 25 RNA-seq libraries. Specifically, we used a high-coverage (∼200 million reads), strand-specific, RNA-seq library generated from mixed whole bodies of apterous *M. persicae* clone O asexual females (Mathers et al. 2017) as well as newly generated (see above) and publicly available (Mathers et al. 2019) unstranded RNA-seq data for *M. persicae* clone O nymphs (derived from apterous asexual females), alate asexual females, apterous asexual females and males (six biological replicates each). All RNA-seq data were trimmed for adapters and low quality bases (quality score < 20) with Trim Golore v0.4.5 (Krueger 2015), retaining reads where both members of the pair are at least 20-bp long. Unstranded RNA-seq data were aligned to the genome with HISAT2 with the parameters “--max-intronlen 25000 --dta-cufflinks” followed by sorting and indexing with SAMtools v1.3 (Li et al. 2009). Strand-specific RNA-seq was mapped as for the unstranded data, with the addition of the HISAT2 parameter “--rna-strandness RF.” We then ran BRAKER2 with UTR training and prediction enabled with the parameters “--softmasking --gff3 --UTR=on.” Strand-specific RNA-seq alignments were split by forward and reverse strands and passed to BRAKER2 as separate BAM files to improve the accuracy of UTR models as recommended in the BRAKER2 documentation. For *A. pisum* clone JIC1, we used unstranded RNA-seq data derived from whole bodies of *A. pisum* clone LSR1 (IAGC 2010) males, asexual females, and sexual females (two biological replicates each) from Jaquiéry et al. (2013). Reads were, trimmed, mapped, and passed to BRAKER2 as for the unstranded *M. persicae* RNA-seq data. Following gene prediction, genes were removed that contained in frame stop codons using the BRAKER2 script getAnnoFastaFromJoingenes.py and the completeness of each gene set was checked with BUSCO using the longest transcript of each gene as the representative transcript.

### X Chromosome Identification

We identified the aphid sex (X) chromosome in our new assemblies of *M. persicae* clone O and *A. pisum* JIC1 based on the ratio of male (M) to asexual female (FA) coverage of Illumina genomic DNA reads. For *M. persicae*, we used whole-genome bisulfite sequencing (BS-seq) reads from Mathers et al. (2019), merging biological replicates by morph. These data are derived from the same clonal population (clone O) as used for the genome assembly. BS-seq reads were aligned to the *M. persicae* clone O v2 genome with Bismark v0.20.0 (Krueger and Andrews 2011) with default parameters. We used Sambamba v0.6.8 to estimate BS-seq read depth in 100-kb fixed windows for M and FA separately using the BAM files generated by Bismark and the parameters “depth window --fix-mate-overlaps --window-size = 100000 --overlap = 100000.” We then calculated the ratio of M to FA read depth per window (i.e., the coverage ratio). Coverage ratios showed scaffold 1 to have the expected X chromosome M to FA coverage ratio (50% that of the autosomes). To generate figure 1*c*, we calculated average M (107×) and FA (82×) coverage excluding scaffold 1 to derive a coverage correction factor for FA (×1.3) and used this to calculate normalized M to FA coverage ratio for each 100 kb window. For *A. pisum* JIC1, we used whole-genome Illumina sequence data of clone AL4 M and FA morphs the from Li et al. (2019). We followed the same procedure as for *M. persicae* clone O with the exception of using BWA-MEM v0.7.17 (Li 2013) to map reads and Sambamba markdup to identify reads derived from polymerase chain reaction duplicates prior to calculating coverage statistics. Scaffold 1 was identified as the X chromosome. Excluding scaffold 1, we calculated average M (45×) and FA (41×) coverage to derive a coverage correction factor for FA (×1.1) and used this to calculate normalized M to FA coverage ratio for each 100-kb window along *A. pisum* JIC1 chromosome-length scaffolds to generate figure 1*d*.

### Reannotation of the Chromosome-Scale Assemblies of *Rhod. prolixus* and *T. rubrofasciata*

We included the recently released chromosome-scale genome assemblies of the blood-feeding hemipterans *Rhod. prolixus* (obtained from the DNA Zoo [https://www.dnazoo.org/, last accessed March 25, 2020; Dudchenko et al. 2017]) and *T. rubrofasciata* (Liu et al. 2019) in our synteny and phylogenomic analyses. The *Rhod. prolixus* chromosome-level assembly has not yet been annotated and we found on initial inspection that the *T. rubrofasciata* gene release is based on the contig assembly of this species and not the chromosome-length scaffolds. We therefore generated de novo gene predictions for these two species using BRAKER2 with evidence from protein alignments created with GenomeThreader v1.7.1 (Gremme et al. 2005). For each species, we soft-masked the genome for known repeats as for *M. persicae* and *A. pisum*. We then ran BRAKER2 with the parameters “--softmasking --gff3 --prg=gth --trainFromGth.” For *Rhod. prolixus*, we used proteins from the original gene release as evidence (Mesquita et al. 2015). For *T. rubrofasciata*, we used proteins from Liu et al. (2019). The final BRAKER2 gene sets for each species were checked completeness using BUSCO as for *M. persicae* and *A. pisum*.

### Phylogenomic Analysis of Sequenced Hemipteran Genomes

We estimated a time calibrated phylogeny of Hemiptera using protein sequences from our new genome assemblies of *M. persicae* clone O and *A. pisum* clone JIC1, the new annotations of the chromosome-scale assemblies of *Rhod. prolixus* and *T. rubrofasciata* and ten previously sequenced Hemiptera: *Myzus cerasi* (Thorpe et al. 2018), *Diuraphis noxia* (Nicholson et al. 2015), *Pentalonia nigronervosa* (Mathers et al. 2020a), *R. maidis* (Chen et al. 2019), *Rhopalosiphum padi* (Thorpe et al. 2018), *Aphis glycines* (version 2) (Mathers 2020), *Bemisia tabaci* MEAM1 (Chen et al. 2016), *Oncopeltus fasciatus* (Panfilio et al. 2019), *Sogatella furcifera* (Wang et al. 2017), and *Nilaparvata lugens* (Xue et al. 2014). Where multiple transcripts of a gene were annotated, we used the longest transcript to represent the gene model. We used OrthoFinder v2.2.3 (Emms and Kelly 2015, 2019) with Diamond v0.9.14 (Buchfink et al. 2015), MAFFT v7.305 (Katoh and Standley 2013), and FastTree v2.1.7 (Price et al. 2009, 2010) to cluster proteins into orthogroups, reconstruct gene trees, and estimate the species tree. The OrthoFinder species tree was rooted according to Johnson et al. (2018). To estimate approximate divergence times for our taxa of interest, we used penalized likelihood implemented in r8s with secondary calibration points derived from Johnson et al. (2018) (supplementary table 9, Supplementary Material online).

### Synteny Analysis

We identified syntenic blocks of genes between *M. persicae*, *A. pisum*, and *R. maidis*, and between *Rhod. prolixus* and *T. rubrofasciata*, using MCScanX v1.1 (Wang et al. 2012). For each comparison, we carried out an all versus all BLAST search of annotated protein sequences using BLASTALL v2.2.22 (Altschul et al. 1990) with the options “-p BlastP - e 1e-10 -b 5 -v 5 -m 8” and ran MCScanX with the parameters “-s10 -b 2,” requiring synteny blocks to contain at least ten consecutive genes and to have a gap of no more than 25 genes. MCScanX results were visualized with SynVisio (https://synvisio.github.io/#/, last accessed March 25, 2020). We parsed the MCScanX results and estimated synonymous and nonsynonymous substitution rates between pairs of syntenic genes using collinearity scripts from Nowell et al. (2018; https://github.com/reubwn/collinearity, last accessed March 25, 2020). We also investigated synteny using orthologous genes identified by OrthoFinder. We performed two additional OrthoFinder runs, one with the chromosome-scale assemblies of *M. persicae*, *A. pisum*, and *R. maidis*, and one using the three aphid assemblies and the chromosome-scale assembly of *Rhod. prolixus*. OrthoFinder was run as described above for the phylogenomic analysis of Hemiptera.

To test for conservation of the X chromosome across Hemiptera, we first identified *Rhod. prolixus* chromosomes that were likely to be homologous to *M. persicae* chromosomes. We therefore mapped their orthologous genes onto chromosomes. Next, we tested for significant enrichment of genes from specific *Rhod. prolixus* (target) chromosomes on each *M. persicae* (focal) chromosome using a binomial test. In each binomial test, the observed ortholog count from a target *Rhod. prolixus* chromosome is the *number of successful trials.* The total number of orthologs on the *M. persicae* focal chromosome is the *total number of trials* (this is equal to the sum of all *Rhod. prolixus* orthologs that map to the focal chromosome). Finally, the *probability of success* is equal to the faction orthologs found on the *Rhod. prolixus* target chromosome, relative to the total number of orthologs. We corrected for multiple testing using the BH procedure (Benjamini and Hochberg 1995). For each focal *M. persicae* chromosome, we also calculated the observed/expected ratio of orthologs from each target *Rhod. prolixus* chromosome. The expected ortholog count was calculated by multiplying the total ortholog count for the focal *M. persicae* chromosome by the faction of all *M. persicae*–*Rhod. prolixus* orthologs found on the target *Rhod. prolixus* chromosome.

### *Myzus persicae* Gene Expression

We investigated patterns of gene expression in the *M. persicae* clone O v2 genome using newly generated (see above) and previously published (Mathers et al. 2019) RNA-seq data for *M. persicae* clone O nymphs (derived from unwinged asexual females), winged asexual females, unwinged asexual females, and winged males (six biological replicates each). Transcript-level expression was estimated for each sample with Kallisto v0.44.0 (Bray et al. 2016) with 100 bootstrap replicates. We identified differentially expressed genes between *M. persicae* morphs using Sleuth (Pimentel et al. 2017), aggerating transcript-level *P* values (Yi et al. 2018). Specifically, we used a likelihood ratio test to identify genes that significantly vary by morph (BH corrected *P* < 0.05). To quantify the magnitude of the change in expression relative to unwinged asexual females (from which the other morphs are derived), we applied pairwise Wald tests between unwinged asexual females and each alternative morph and recorded the effect size (beta) which approximates the log_2_ fold change in expression. We considered genes to be “morph biased” if they had a significant likelihood ratio test result and abs. beta > 0.5 in any morph relative to unwinged asexual females. To identify genes that were specifically upregulated in males, we identified the subset of “morph-biased” genes that had beta > 0.5 in winged males and beta < 0.5 in winged asexual females and nymphs.

To test for dosage compensation in *M. persicae* clone O, we calculated the log_2_ ratio of winged male to unwinged asexual female gene expression using transcripts per million (TPM) expression values estimated by Kallisto for all genes with expression of at least one TPM in both morphs. For each gene, we used the longest transcript to represent the gene. We then compared expression ratios for genes on the X chromosome and the autosomes with a Wilcoxon rank-sum test.

### TE Analysis

To investigate the distribution of TEs in *M. persicae* clone O v2 and *A. pisum* JIC1 v1, we generated a comprehensive TE annotation. For each assembly, we modeled TEs de novo with RepeatModeler v1.0.8 (Smit and Hubley 2008) and then merged the de novo repeats with known repeats from the RepBase Insecta library (Bao et al. 2015) using ReannTE_MergeFasta.pl (https://github.com/4ureliek/ReannTE). We then annotated TEs across each genome with RepeatMasker v4.0.7 (Smit et al. 2005; Tarailo-Graovac and Chen 2009) using the species-specific merged TE library. We calculated TE density in 100 kb and 1 Mb fixed windows with DensityMap (Guizard et al. 2016), grouping all TEs together, and also separately for DNA transposons, LINEs, LTR retrotransposons, rolling-circle transposons, and short interspersed nuclear elements. We also calculated the density of expressed genes in the same windows. For *M. persicae*, we used genes classified as expressed by sleuth (estimated count >4 in at least 12/24 samples) in the “morph-biased” expression analysis (above). To generate equivalent data for *A. pisum*, we ran Kallisto and Sleuth as for the *M. persicae* morph-biased expression analysis (above) using RNA-seq data derived from whole bodies of *A. pisum* clone LSR1 (IAGC 2010) males, asexual females, and sexual females (two biological replicates each) from Jaquiéry et al. (2013). Genes were considered expressed if they had an estimated read count >4 in at least three out of six samples.

We investigated the repeat content of autosomal synteny blocks and autosomal synteny breakpoint regions in *M. persicae* clone O v2 and *A. pisum* JIC1 v1 using BEDTools v2.28.0 (Quinlan and Hall 2010) and the TE annotations described above. We defined synteny breakpoint regions as the gaps between synteny blocks identified by MCScanX analysis of *M. persicae* clone O v2 and *A. pisum* JIC1 v1 (see Synteny Analysis). The genomic coordinates of synteny blocks were defined based on the start position of the first gene and the end position of the last gene in each block. We then identified the genomic coordinates of synteny breakpoint regions using BEDTools complement (i.e., we identified all regions in between autosomal synteny blocks). We excluded chromosome ends as they may or may not correspond to breakpoint regions and may contain repetitive (sub)telomeric sequence that would bias our analysis (i.e., breakpoint regions had to be flanked by a synteny block at either end). As synteny blocks were defined based on the locations of homologous genes (rather than sequence alignments) and allow gaps of up to 25 genes within blocks, our analysis should not be affected by the breakup of synteny blocks by lineage-specific TE accumulation within otherwise syntenic genomic regions. TEs overlapping synteny blocks and breakpoint regions were identified using BEDTools intersect and we recorded the span (in bp) and count of TEs by class (i.e., summing independently for DNA, LINE, LTR, rolling-circle, SINE, and unclassified TEs). To test for significant enrichment of TEs within synteny breakpoint regions, we simulated 10,000 sets of random regions, each with the same size distribution as the observed synteny breakpoint regions, and repeated the analysis. *P* values for each TE class were determined based on the number of simulated regions with a TE count equal to or greater than the TE count of the same class in the observed synteny breakpoint regions divided by the number of simulations (*n* = 10,000). Additionally, for each TE class, we calculated the expected span in bp within autosomal synteny breakpoint regions based on the total size of the autosomal synteny breakpoint regions and the autosome-wide TE proportion of each class and compared this with the observed value. These analysis were carried out independently using both *M. perisicae* clone O v2 and *A. pisum* JIC1 v1 as the reference.

To generate TE age distributions for *M. persicae* clone O v2 and *A. pisum* JIC1, we ran RepeatMasker separately for the autosomes and the X chromosome for each species and parsed the output with parseRM_GetLandscape.pl (https://github.com/4ureliek/Parsing-RepeatMasker-Outputs, last accessed March 25, 2020). We used the CpG adjusted Kimura 2-parameter distance of each TE insertion from its corresponding consensus sequence as a proxy for TE age.

## Supplementary Material

Supplementary data are available at *Molecular Biology and Evolution* online.

## Supplementary Material

msaa246_Supplementary_Data
